# Outcomes and Healthcare Resource Utilisation in Adults With von Willebrand Disease Receiving On‐Demand Recombinant von Willebrand Factor in the United Kingdom

**DOI:** 10.1111/ejh.70032

**Published:** 2025-10-10

**Authors:** Mike Laffan, Heena Howitt, Cheryl Jones, Sarah Brighton, Rosa Willock, Anna Sanigorska, Oliver Heard

**Affiliations:** ^1^ Centre for Haematology Imperial College London, Hammersmith Hospital London UK; ^2^ Takeda UK Ltd. London UK; ^3^ HCD Economics UK

**Keywords:** adult, delivery of healthcare, health resources, retrospective studies, treatment outcome, United Kingdom, von Willebrand diseases, von Willebrand factor

## Abstract

**Objectives:**

We describe treatment outcomes and healthcare resource utilisation (HCRU) in adults with von Willebrand disease (VWD) treated on demand with recombinant von Willebrand factor (rVWF) in the United Kingdom.

**Methods:**

Retrospective chart review of adults (≥ 18 years) with congenital VWD receiving first‐time rVWF for the on‐demand treatment of spontaneous/traumatic bleeds, or the prevention/treatment of surgical bleeds, between 1 October 2020 and 30 June 2022 at seven hospitals. Treatment, outcome and VWD‐related HCRU data associated with the on‐demand treatment of all recorded bleeds were collected at first (index) treatment, for 12 months pre‐index and 3–12 months post‐index.

**Results:**

Twelve patients (91.7% female and White/Caucasian, mean age 34.2 years) were treated on demand with rVWF at index: bleed resolution was achieved for all bleeds with limited requirement for additional treatments, no treatment switches, and no complications reported. Physician‐rated treatment satisfaction was ‘excellent’ (82.6%) or ‘good’ (17.4%) for all recorded on‐demand rVWF‐treated bleeds (*n* = 23). Patients receiving any on‐demand treatment required an inpatient admission and ≥ 1 outpatient visit in 33.7% and 66.7% of cases, respectively.

**Conclusions:**

Results support the effectiveness and safety profile of on‐demand rVWF treatment for spontaneous/traumatic bleeds in adults with VWD, adding to the growing body of evidence for rVWF.

## 1 Introduction

Von Willebrand disease (VWD) is considered the most common inherited bleeding disorder, both among males and females [1]. In 2020, it was estimated that 16.5 per 100 000 people in the UK had VWD (type 1: 7.2 per 100 000 people; type 2: 2.5 per 100 000; type 3: 0.3 per 100 000; remaining patients ‘unclassified’ or ‘low von Willebrand factor’ [VWF]) according to the UK National Haemophilia Database (NHD) [2]. Patients with VWD most commonly experience spontaneous mucosa‐associated bleeding (e.g., epistaxis, menorrhagia, or oral/gum bleeding) and bleeding after surgery or trauma [3, 4, 5]. Evidence suggests that patients with VWD have greater healthcare resource utilisation (HCRU) and poorer health‐related quality of life than the general population [6, 7, 8, 9, 10, 11].

In the United Kingdom (UK), recombinant von Willebrand factor (rVWF; vonicog alfa) is indicated for the prevention and treatment of haemorrhage or surgical bleeding in adults with VWD, when desmopressin treatment alone is ineffective or is contraindicated [12]. In Phase 3 clinical trials, rVWF has been shown to be efficacious for the on‐demand and peri‐operative management of bleeding in patients with VWD [13, 14].

Since rVWF was licensed for adults with VWD in the UK in August 2018, limited analyses of real‐world data describing its use in clinical settings have been undertaken. Therefore, further information on rVWF treatment outcomes in patients with VWD in real‐world UK settings is needed to inform future decision‐making with regard to the management of these patients. Furthermore, there is a paucity of real‐world evidence to describe HCRU among patients with VWD who are treated with rVWF in the UK.

This retrospective chart review evaluated real‐world experience with rVWF used on demand to treat spontaneous or traumatic bleeds, or for the prevention and resolution of bleeds during surgery, in the UK. The objective of this analysis is to describe treatment outcomes and HCRU in adults with congenital VWD treated on demand with rVWF. Outcomes and HCRU associated with the use of rVWF in adults with VWD in surgical settings will be reported in a parallel publication [15].

## 2 Methods and Materials

### 2.1 Study Design

**FIGURE 1:**
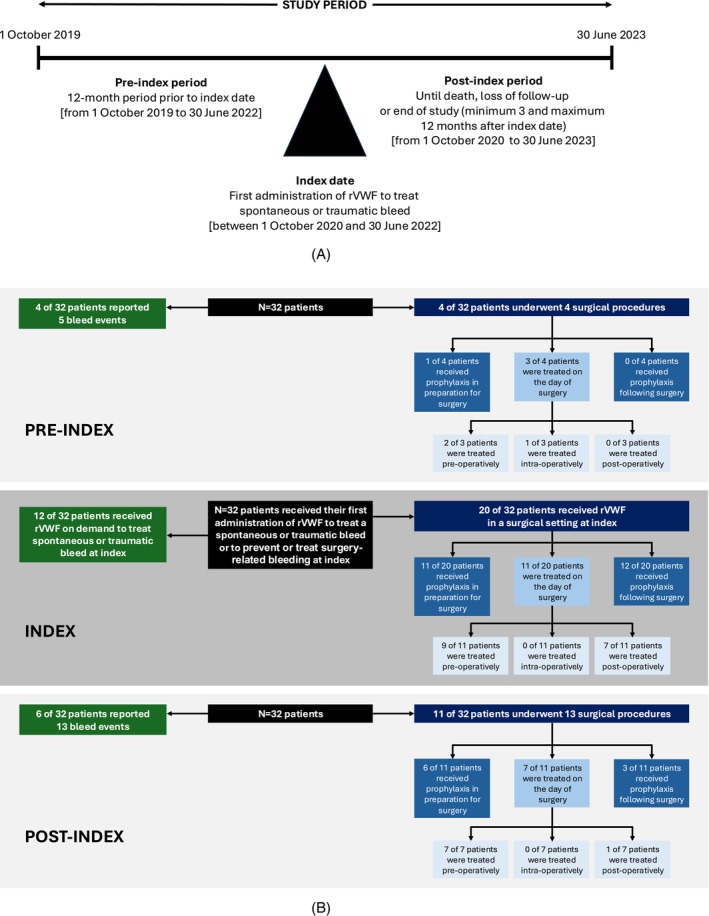
(A) Study design and (B) patient flow diagram: Index and pre‐/post‐index (*N* = 32). rVWF, recombinant von Willebrand factor.

A retrospective chart review study was conducted at seven UK hospitals. Patients were enrolled at the time of their first ever rVWF administration (index date) between 1 October 2020 and 30 June 2022 (index period) (Figure 1A). Patient data were collected from medical records using an electronic case report form at index treatment, for 12 months pre‐index, and until death, loss of follow‐up, or end of study (3–12 months post‐index) by the treating physician. This study was conducted in accordance with Good Clinical Practice. Full ethical approval was obtained from the Yorkshire and Humber—Leeds West Research Ethics Committee, and informed consent was collected prior to patient enrolment.

### 2.2 Patient Population

Eligible patients with a diagnosis of congenital VWD must have received their first ever dose of rVWF within its licensed indication, either on demand to treat a spontaneous or traumatic bleed or to prevent or treat surgery‐related bleeding, during the index period [12]. Eligible patients had to be at least 18 years of age at the time of their index dose and have at least 3 months of follow‐up after their index administration. Patients were excluded if they had other bleeding disorders or factor deficiency (including acquired von Willebrand syndrome), VWF neutralising antibodies/inhibitors, or participated in clinical trials during the study period. Two adults, one with a muscle haematoma and one who underwent minor surgery, were eligible for inclusion in the study but have been excluded from this analysis because they received long‐term VWF prophylaxis during the study period.

### 2.3 Objectives

The primary objective of this analysis was to describe the real‐world first‐time on‐demand use of rVWF for the treatment of spontaneous or traumatic bleeds at index, including subsequent bleed‐related outcomes, in adults with congenital VWD. The secondary objectives were to describe VWD‐related HCRU associated with all spontaneous or traumatic bleeds recorded during the entire study period, and the type, severity, treatment, treatment duration, and related outcome(s) of all spontaneous or traumatic bleeds recorded during the 12 months prior to and the 3–12 months following the index administration of rVWF.

Exploratory objectives included describing HCRU by type of hospitalisation for bleeds treated on demand with or without rVWF; differences in on‐demand treatment and bleed‐related outcomes for bleed events treated with rVWF only, rVWF plus another treatment, other factor treatments, and treatment involving a switch; and characteristics, treatment, outcomes, and HCRU of bleed events treated on demand with rVWF stratified by age group, sex, VWD type, time since diagnosis, number of comorbidities, and bleed. Exploratory objectives used pooled data from all study periods.

### 2.4 Assessments

Treatment and outcome data collected included the number of patients and bleed events for which on‐demand rVWF was administered across the entire study period, including type (epistaxis; muscle haematoma; oral, gastrointestinal [GI], central nervous system [CNS], or pregnancy‐related bleed; menorrhagia or trauma [not treated with surgery]) and severity (mild, moderate or severe, as categorised by the treating physician). Additional data on the on‐demand treatment of bleeds (including number of infusions received by the patient, average dose/kg, total dose received and treatment duration for rVWF plus any other drugs used) were also collected. Other drugs used included one or more of plasma‐derived (pd)VWF concentrates, factor VIII (FVIII) products, desmopressin, or tranexamic acid (TXA). It should be noted that multiple on‐demand treatments administered to treat the same bleed (i.e., treatment combinations) cannot be interpreted as simultaneous use of the products, as the exact timing of treatment administration was not recorded. Information on treatment switches, including the reason for switching, was also collected. Bleed control (i.e., the bleed is brought under control and is expected to stop [assessed as yes, no, or partial]) and bleed resolution (i.e., the bleed stopped completely after treatment [assessed as yes or no]) data were described where applicable, according to the opinion of the treating physician.

Physician‐assessed satisfaction with rVWF treatment was collected using a four‐point rating scale (‘excellent’, ‘good’, ‘moderate’ or ‘poor’); and adverse events and treatment‐emergent adverse events were collected using the Common Terminology Criteria for Adverse Events grading version 5 (mild, moderate, severe, life‐threatening and death) [16].

VWD‐related HCRU data collected comprised the number of patients requiring inpatient and intensive care unit [ICU] admissions, the duration of hospital stays (inpatient and ICU), and the number of outpatient visits (day cases).

### 2.5 Statistical Analyses

All data were summarised descriptively. Continuous variables were summarised as means and standard deviation (SD) of the mean; categorical variables were presented as frequencies and percentages. Endpoints were reported separately for the index bleed and any pre‐ or post‐index bleeds during the follow‐up period. Additional analyses described treatment, outcomes, and HCRU associated with on‐demand rVWF treatment of spontaneous or traumatic bleeds stratified by age group, sex, VWD type, time since diagnosis, number of comorbidities, and bleed severity (mild, moderate, or severe). Analyses were performed using STATA 17 (StataCorp. 2021. Stata Statistical Software: Release 17. College Station, TX: StataCorp LLC).

## 3 Results

In total, 32 patients received rVWF to treat a spontaneous or traumatic bleed or to prevent or treat a surgery‐related bleed at index (Figure 1B and Table S1).

### 3.1 On‐Demand Treatment of Spontaneous or Traumatic Bleeds With rVWF at Index

#### 3.1.1 Demographics and Baseline Characteristics of Patients

Overall, rVWF was used on demand to treat a spontaneous or traumatic bleed at index in 12 patients with a mean (SD) age at index of 34.2 (10.6) years and the time since diagnosis was 13.6 (10.6) years. Most of these patients were female (91.7%) and White/Caucasian (91.7%) (Table 1). The mean (SD) weight and body mass index of these patients were 81.3 (16.1) kg and 28.6 (6.6) kg/m^2^, respectively. Two patients had type 1 and 10 patients had type 2 VWD, five patients had a familial history of VWD, and 33.3% of patients had at least one comorbidity at index. Laboratory test values at presentation by VWD type and by bleed severity are reported in Table S2.

**TABLE 1:** Demographics and baseline characteristics of adults with VWD receiving on‐demand rVWF to treat a spontaneous or traumatic bleed at index.

Characteristic	*N* = 12
Age at index (years), mean (SD)	34.2 (10.6)
Age groups at index, *n* (%)	
18–29 years	4 (33.3)
30–44 years	6 (50.0)
45–64 years	2 (16.7)
Female, *n* (%)	11 (91.7)
White/Caucasian, *n* (%)	11 (91.7)
Weight (kg), mean (SD)	81.3 (16.1)
BMI (kg/m^2^), mean (SD)	28.6 (6.6)
VWD type, *n* (%)	
Type 1	2 (16.7)
Type 2A	3 (25.0)
Type 2B	4 (33.3)
Type 2M	3 (25.0)
Age at diagnosis (years), mean (SD)	20.1 (14.0)
Time since diagnosis (years), mean (SD)	13.6 (10.6)
Familial history of VWD, *n* (%)	5 (41.7)
Unknown, *n* (%)	4 (33.3)
Family member with VWD, *n* (%)^a^	
Parent	4 (80.0)
Sibling	1 (20.0)
Grandparent	3 (60.0)
Other	2 (40.0)
No history of GI bleeding, *n* (%)	12 (100.0)
Laboratory test values at presentation (IU/mL)^b^, mean (SD)	
VWF:RCo [*n* = 7]	0.4 (0.3)
FVIII:C [*n* = 8]	1.1 (0.8)
VWF:Ag [*n* = 8]	0.8 (0.8)
FVIII < 0.4 at index bleed presentation^b^, *n* (%), [*n* = 8]	1 (12.5)
Comorbidities at index^c^, *n* (%)	
0	8 (66.7)
1	3 (25.0)
2	1 (8.3)
Comorbidities at index by type^d^, *n* (%)	
Disease of digestive system	1 (8.3)
Endocrine, nutritional or metabolic disease	1 (8.3)
Mental or behavioural disorder	1 (8.3)
Neurological	1 (8.3)
Obstetrics/gynaecology	1 (8.3)

*Note: N* = number of patients.

Abbreviations: Ag, antigen; BMI, body mass index; FVIII, factor VIII; GI, gastrointestinal; RCo, ristocetin cofactor; rVWF, recombinant von Willebrand factor; SD, standard deviation; VWD, von Willebrand disease; VWF, von Willebrand factor.

^a^ Percentage calculated with number of patients with non‐missing data in denominator.

^b^ Not all lab tests were performed at presentation on all patients. Mean (SD) is reported only for patients on whom each test was performed [*n* = *x*].

^c^ Within 2 years of data abstraction.

^d^ At least one comorbidity in category.

#### 3.1.2 Bleed Characteristics and Treatment

Five patients (41.7%) had pregnancy‐related bleeds, two (16.7%) had a muscle haematoma, and one each (8.3%) had epistaxis, an oral bleed, a GI bleed, menorrhagia, and trauma (not treated with surgery). Two‐thirds of bleeds (66.7%) were classified by the treating physician as mild: of the remainder, two (16.7%) were classified as moderate and two were classified as severe.

Nine patients (75.0%) received rVWF without any additional FVIII for the treatment of their index bleed: four were treated with rVWF alone and five were treated with rVWF in combination with TXA (Tables 2 and S3). Two patients (16.7%) were treated with pdVWF/FVIII complex in addition to rVWF at their index bleed: one patient on the same day while the other patient switched from pdVWF/FVIII complex to rVWF after a single infusion. One patient (8.3%) switched from rFVIII to rVWF at their index bleed. TXA was also administered in all three of these cases.

**TABLE 2:** On‐demand treatment of spontaneous and traumatic bleeds at index in adults with VWD by treatment.

Medication	rVWF	pdVWF/FVIII complex	rFVIII	Tranexamic acid
Variable				
Number of bleed events, *n* (%)^a^	12 (100.0)	2 (100.0)	1 (100.0)	8 (100.0)
Dose (IU or mg), mean (SD)	2487.6 (1205.7)	2000.0 (0.0)	3000.0	1000 (0.0)
Number of infusions	2.3 (2.6)	1.0 (0.0)	1	29.8 (39.4)
Dose per kg per infusion	30.7 (13.6)	26.7 (1.5)	25.9	12.5 (2.9)
Total consumption (IU or mg)	4870.8 (4443.9)	2000.0 (0.0)	3000.0	29750.0 (39394.5)
Duration of treatment (days)	1.8 (1.3)	1.0 (0.0)	1.0	9.8 (13.2)

Abbreviations: FVIII, factor VIII; pd, plasma‐derived; rFVIII, recombinant factor VIII; rVWF, recombinant von Willebrand factor; SD, standard deviation; VWD, von Willebrand disease; VWF, von Willebrand factor.

^a^ Patients could receive more than one treatment on demand for a bleed, either in combination or after switching.

The 12 patients who received on‐demand rVWF at index had a mean (SD) 2.3 (2.6) infusions per patient. The mean (SD) dose per infusion was 30.7 (13.6) IU/kg, the mean (SD) total consumption was 4870.8 (4443.9) IU, and the mean (SD) treatment duration was 1.8 (1.3) days (Table 2).

#### 3.1.3 Bleed Outcomes

All 12 patients who received on‐demand rVWF at index achieved bleed control and bleed resolution. Two of these patients who had received pdVWF/FVIII complex did not reach bleed resolution with this treatment. One of these patients, who was treated with rVWF on the same day as pdVWF/FVIII, achieved bleed control. The other patient was switched to rVWF and subsequent TXA treatment, although the reason for the switch was not specified. One patient who received rFVIII and TXA did not achieve bleed resolution or control with this treatment and was switched to rVWF and TXA treatment due to lack of efficacy/poor half‐life.

#### 3.1.4 Safety

No adverse events related to the treatment of spontaneous or traumatic bleeds at index were reported during the study period.

### 3.2 On‐Demand Treatment of Spontaneous or Traumatic Bleeds Pre‐ and Post‐Index

#### 3.2.1 Bleed Characteristics, Treatment and Outcomes

During the 12 months prior to their first rVWF administration (pre‐index), four of the 32 patients in the study reported a spontaneous or traumatic bleed, with one patient reporting two bleed events, all of which were mild. These were two muscle haematomas, two traumas (not treated with surgery), and one oral bleed. Three of these bleed events were treated with pdVWF/FVIII complex, two of which were also treated with TXA, and two bleed events were not treated (Table S3). Details of drug usage by product for pre‐index bleeds are reported in Table S4. Bleed control and resolution were achieved for all three pre‐index bleeds treated with pdVWF/FVIII complex. There were no reported treatment switches pre‐index.

During the 3–12 months after their first rVWF administration (post‐index), six of the 32 patients in the study reported a total of 13 bleed events. The majority (61.5%) were mild, 23.1% were moderate, and 15.4% were severe. Five traumas (not treated with surgery) were reported post‐index, along with four menorrhagia, two epistaxes, one CNS‐ and one pregnancy‐related bleed. On‐demand rVWF was used post‐index to treat 11 of the 13 bleed events (Table S3). Ten were initially treated with rVWF (two with rVWF only; five with rVWF + TXA; two with rVWF, pdVWF/FVIII complex + TXA; and one with rVWF + rFVIII + TXA) and there was one switch from pdVWF/FVIII complex to rVWF. One bleed was treated with pdVWF/FVIII complex + TXA, and one bleed was not treated. The mean (SD) number of rVWF infusions per patient was 2.2 (3.3), the mean (SD) dose per infusion was 35.0 (11.4) IU/kg, the mean (SD) total consumption was 4786.4 (4294.7) IU, and the mean (SD) treatment duration was 1.6 (1.5) days (Table 3).

**TABLE 3:** **On**‐demand treatment of spontaneous and traumatic bleeds post‐index (*N* = 13) in adults with VWD by treatment.

Medication	rVWF	pdVWF/FVIII complex	FVIII	Tranexamic acid
Variable				
Number of bleed events, *n* (%)	11 (100.0)	4 (100.0)	1 (100.0)	9 (100.0)
Dose (IU or mg), mean (SD)	2747.7 (1061.8)	1841.7 (641.4)	3000.0	1000.0 (0.0)
Number of infusions	2.2 (3.3)	1.5 (1.0)	1.0	50.9 (39.0)
Dose per infusion (IU/kg or mg/kg)	35.0 (11.4)	29.2 (9.5)	48.4	13.9 (2.7)
Total consumption (IU or mg)	4786.4 (4294.7)	3175.0 (3233.5)	3000.0	50888.9 (38956.5)
Duration of treatment (days)	1.6 (1.5)	1.3 (0.5)	1.0	13.1 (9.4)

Abbreviations: FVIII, factor VIII; pd, plasma‐derived; rVWF, recombinant von Willebrand factor; SD, standard deviation; VWD, von Willebrand disease; VWF, von Willebrand factor.

Post‐index, eight of the 11 bleeds treated on demand with rVWF achieved bleed resolution (one bleed had an unknown bleed resolution outcome), nine achieved bleed control, and two achieved partial bleed control with no reported treatment switches from rVWF to an alternative treatment. Details of bleed outcomes by product for all post‐index bleed events are reported in Table S5.

### 3.3 Satisfaction With On‐Demand rVWF Treatment of Bleed Events

For all bleed events treated on‐demand with rVWF (12 at index and 11 during the post‐index period), the treating physicians rated treatment satisfaction as ‘excellent’ (82.6%) or ‘good’ (17.4%).

### 3.4 VWD‐Related HCRU Associated With On‐Demand Treatment of Bleed Events

An overview of VWD‐related HCRU for bleed events during all study periods (index, pre‐ and post‐index) is presented in Table 4. At index, inpatient admissions and/or outpatient visits were required by 11 patients (91.7%) treated on demand for spontaneous or traumatic bleeds, none of which included admission to an ICU (Table 4). Six patients (50.0%) had an inpatient admission, with a mean (SD) length of stay of 3.3 (1.0) days. Five patients (41.0%) had a single outpatient visit at index. During the 12 months pre‐index, three patients (60.0%) had a single outpatient visit; there were no inpatient admissions. Post‐index, all 13 patients required an inpatient admission and/or outpatient visit. Four patients (30.8%) had an inpatient admission, with a mean (SD) length of stay of 3.3 (2.5) days. Ten patients (76.9%) had an outpatient visit, with mean (SD) 1.1 (0.3) outpatient visits per patient. One patient had both an inpatient admission and an outpatient visit.

**TABLE 4:** VWD‐related HCRU of adults receiving on‐demand treatment of spontaneous or traumatic bleeds.

Time point/period	At index (*N* = 12 bleeds)	12 months pre‐index (*N* = 5 bleeds)	3–12 months post‐index (*N* = 13 bleeds)
Variable			
Number of adults with an inpatient admission, *n* (%)	6 (50.0)	0 (0.0)	4 (30.8)
Length of inpatient stay (days), mean (SD)	3.3 (1.0)	N/A	3.3 (2.5)
Number of adults with an ICU admission, *n* (%)	0 (0.0)	0 (0.0)	0 (0.0)
Length of ICU stay (days), mean (SD)	N/A	N/A	N/A
Number of adults with an outpatient visit (day cases), *n* (%)	5 (41.7)	3 (60.0)	10 (76.9)
Number of outpatient visits per patient, mean (SD)	1.0 (0.0)	1.0 (0.0)	1.1 (0.3)

Abbreviations: HCRU, healthcare resource utilisation; ICU, intensive care unit; N/A, not applicable; rVWF, recombinant von Willebrand factor; SD, standard deviation; VWD, von Willebrand disease.

### 3.5 Exploratory Outcomes

#### 3.5.1 VWD‐Related HCRU for Pooled Bleed Events Treated With or Without rVWF by Type of Hospitalisation

**FIGURE 2:**
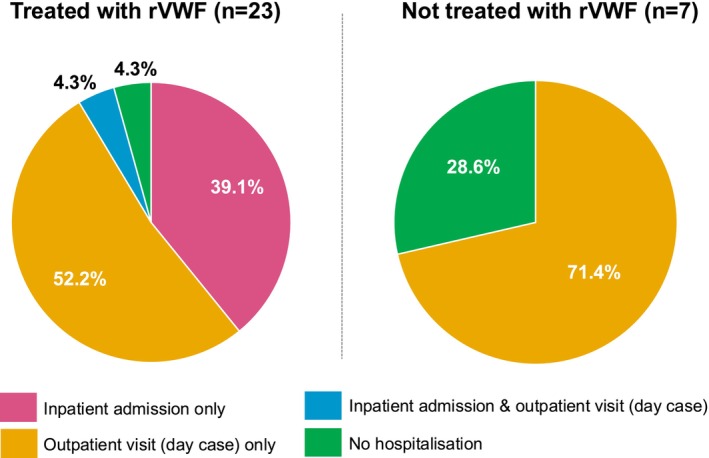
HCRU associated with the on‐demand treatment of spontaneous and traumatic bleeds with or without rVWF* in adults with VWD (pooled across index, pre‐ and post‐index periods). *Bleeds treated without rVWF may have been treated with pdVWF/FVIII complex, pdVWF/FVIII complex + TXA, or remained untreated. FVIII, factor VIII; HCRU, healthcare resource utilisation; pd, plasma‐derived; rVWF, recombinant von Willebrand factor; TXA, tranexamic acid; VWD, von Willebrand disease; VWF, von Willebrand factor.

Pooled data from all treatment periods (rVWF was excluded from the pre‐index period) revealed that 23 out of a total of 30 bleeds (76.7%) were treated with rVWF (of which 60.8% were mild, 21.7% were moderate and 17.4% were severe). Seven mild bleeds (23.3%) were treated with products not including rVWF (Figure 2). Nine (39.1%) bleeds treated with rVWF required an inpatient admission only and had a higher mean (SD) consumption of rVWF than 12 (52.2%) of bleeds that required an outpatient visit only; 6422.2 (6047.2) versus 4008.3 (2491.9) IU per patient, respectively. The mean (SD) dose per kg per patient was lower for inpatients (26.0 [9.4] IU/kg) compared with outpatients (36.3 [13.5] IU/kg); however, those patients requiring an inpatient admission were treated over a longer duration (mean [SD] 2.6 [1.9] vs. 1.3 [0.5] days, respectively).

#### 3.5.2 Further Exploratory and Stratified Analyses

Differences in on‐demand treatment and bleed‐related outcomes for pooled index, pre‐ and post‐index bleed events treated with rVWF only, or with rVWF plus another treatment are described in Section S1. Characteristics, treatment, outcomes and HCRU of bleed events treated on demand with rVWF stratified by age group, sex, VWD type, time since diagnosis, number of comorbidities and bleed severity are described in Section S2.

## 4 Discussion

This chart review provides real‐world evidence of the efficacy of on‐demand rVWF for the treatment of spontaneous and traumatic bleeds in line with data from a clinical trial [13], which demonstrates the replicability of these outcomes. We believe this study to be the largest of its kind in the UK. Overall, the study showed that rVWF is effective for the on‐demand treatment of bleed events in adults with type 1 or type 2 VWD, as bleed resolution was achieved for all spontaneous or traumatic bleeds treated on demand with rVWF at index. The limited requirement for additional treatments (e.g., FVIII) to be used in conjunction with rVWF to resolve bleeding further emphasised the effectiveness of the treatment. Only one patient was treated for their index bleed with rVWF plus TXA in combination with another factor treatment, while two switched from another factor treatment to rVWF plus TXA; the remaining nine patients received rVWF (five with TXA) without any additional FVIII. This real‐world evidence also supports the safety profile of rVWF, with no complications reported with on‐demand rVWF treatment.

A similar study conducted using the same methodology across Europe (Austria, Denmark, France, Germany, the Netherlands and Sweden) also reported that bleed resolution was achieved for all spontaneous or traumatic bleeds treated on demand with rVWF at index (except one of unknown bleed resolution outcome) [17], mirroring the results of this UK‐based chart review.

Furthermore, in the current study, most (72.7%) post‐index bleed events were resolved with rVWF, two (18.1%) were reported not to have achieved bleed resolution, and one had an unknown bleed resolution outcome. Notably, no treatment switches from rVWF to an alternative treatment were described, and physician‐rated treatment satisfaction was rated as ‘excellent’ or ‘good’ for all bleed events treated with rVWF. This aligns with outcomes observed in a pivotal Phase 3 clinical trial of on‐demand treatment with rVWF, in which one infusion with rVWF together with rFVIII was reported as an effective treatment combination for 81.8% of bleeds [13].

Overall, 68.8% of the total study population was female and 91.7% of the patients treated on demand with rVWF at index were female. This gender skew may be a result, at least in part, of the bleed types treated. At index, 6 of the 12 bleeds treated on demand were female sex‐specific (i.e., pregnancy‐related or menorrhagia) and 5 of 18 pre‐ and post‐index bleeds treated on demand were also female sex‐specific.

Two‐thirds of bleeds at index were mild, with the remainder evenly divided between moderate and severe. The recommended dose of rVWF for minor haemorrhages is 40–50 IU/kg at initial dose and 40–50 IU/kg every 8 to 24 h (or as long as deemed clinically necessary) [12]. For major haemorrhages, an initial dose of 50–80 IU/kg is recommended, followed by 40–60 IU/kg every 8 to 24 h for approximately 2–3 days (or as long as deemed clinically necessary). However, the mean rVWF dose per infusion for bleeding episodes in the current study was 30.7 IU/kg—lower than licensed recommendations [12], suggesting a difference in real‐world utilisation versus clinical trials. Although this average included two patients treated with a 650 IU dose, which equated to 7.6 and 8.9 IU/kg, removing these outliers still resulted in a mean dose below 40 IU/kg.

Importantly, results from the stratified analyses demonstrated the variability of rVWF dosing across different patient characteristics, such as VWD type and sex, and bleed characteristics (see Supporting Information). For example, higher doses were associated with increasing bleed severity. These results highlight the importance of assessing the context and characteristics of each bleed event and the individual needs of the patient in order to provide the most appropriate regimen to resolve a spontaneous or traumatic bleed.

Existing evidence suggests that HCRU is higher among people with VWD than in the general population. Holm et al. determined that subjects with VWD had a considerably higher consumption of healthcare resources, including twice as many hospitalisations compared with controls in a study of national registers in Sweden [9]. Moreover, Lu et al. found that major bleeding events in patients with VWD were associated with increased HCRU and costs, mostly inpatient costs due to more admissions and longer stays compared with patients without major bleeding in the USA [11].

To our knowledge, the results of the current study are the first to describe real‐world evidence of HCRU in UK patients with VWD who received rVWF on demand to treat spontaneous and traumatic bleeds. We found the on‐demand treatment of bleeds with any treatment in patients with VWD required an inpatient admission in 37.0% of cases and at least one outpatient visit in 66.7% of cases across all study periods. Interestingly, the inpatient attendance and outpatient visit rates at index rVWF administration to treat spontaneous or traumatic bleeds (39.1% and 52.2% respectively, with one patient having both an inpatient admission and an outpatient visit) were different from those observed in Sun et al.'s European study [17], which found rates of 62% and 39%, respectively [18]. It could be hypothesised that the lower rate of inpatient admissions observed in the UK study, compared with the European study, was due to the timing of the two studies (European study: January 2019 to October 2020; UK study: October 2019 to June 2023) and the impact of the COVID‐19 pandemic (from spring 2020) on hospital capacity and the preference for inpatient stays. More research is required to assess HCRU in patients with VWD across treatment centres both in and outside the UK to provide stakeholders with HCRU estimates to inform future decision‐making.

The study design allowed the inclusion of patients across a diverse population with a range of bleed types, thereby providing evidence to support the versatility of rVWF use in various real‐world contexts, while maximising the available sample size to facilitate as much confidence as possible in the study results. Despite this approach, the bleed event sample size was a limitation. The study index period from 1 October 2020 and 30 June 2022 coincided directly with the COVID pandemic in the UK, as well as the multiple lockdowns, and therefore likely contributed to the relatively small number of patients eligible for inclusion in the study. Furthermore, the bleed events were separated across three time periods (index, pre‐ and post‐index) resulting in small numbers of patients in each period; therefore, outliers may have considerably skewed the mean results presented, for example, in terms of dose or treatment duration, as a result. Furthermore, the limited sample size prohibited further stratification of the results by bleed type. As a result of the COVID pandemic, it is also possible that patients only sought assistance for more severe bleeds during this time, with a bias towards greater use of a DDAVP‐only treatment approach outside expert centres. It should be noted, all adults with VWD treated on demand with rVWF in this study had type 1 or 2 VWD, and most were female, limiting the generalisability of these results to type 3 VWD and male patients; therefore, further research is needed to address treatment patterns and outcomes for male patients and those with type 3 VWD. As a retrospective chart review, this study is subject to inherent limitations, including missing data and potential inconsistencies in documentation. Furthermore, bleed severity, bleed control, bleed resolution and physician satisfaction were all subjective assessments and therefore subject to the inherent limitations of retrospective studies.

## 5 Conclusions

The results of this real‐world UK chart review support the effectiveness and safety profile of on‐demand rVWF for the treatment of spontaneous and traumatic bleeds in adults with type 1 and type 2 VWD, adding to the growing body of published evidence on rVWF in VWD. This study also provides valuable insights into how physicians prescribe and administer on‐demand rVWF to their patients in clinical practice in the real world, and how their practice may differ from clinical trials and/or licensed recommendations.

## Author Contributions

Mike Laffan made substantial contributions to the conception and design of the work and acquisition of data for the work, and critically revised the work for important intellectual content. Heena Howitt made substantial contributions to the conception and design of the work and interpretation of data for the work, and critically revised the work for important intellectual content. Cheryl Jones made substantial contributions to the conception and design of the work and the analysis of data for the work, critically revised the work for important intellectual content. Sarah Brighton made substantial contributions to the analysis and interpretation of data for the work, and critically revised the work for important intellectual content. Rosa Willock made substantial contributions to the conception and design of the work and analysis of data for the work, and critically revised the work for important intellectual content. Anna Sanigorska made substantial contributions to the analysis of data for the work, and critically revised the work for important intellectual content. Oliver Heard made substantial contributions to the interpretation of data for the work and critically revised the work for important intellectual content. All authors approved the final version to be published and agreed to be accountable for all aspects of the work in ensuring that questions related to the accuracy or integrity of any part of the work are appropriately investigated and resolved.

## Conflicts of Interest

Mike Laffan has received grant/research/speaker fees from LEO Pharma, Takeda, Pfizer, Roche‐Chugai, Sobi, AstraZeneca, and BioMarin, has acted as a Consultant for AstraZeneca on BMN331201 HAE gene therapy. Heena Howitt and Oliver Heard were employees of Takeda UK Ltd. at the time of this study; they are now ex‐employees. Sarah Brighton, Cheryl Jones, Rosa Willock, and Anna Sanigorska were employees of HCD Economics, an organisation paid by Takeda UK Ltd. for contracted research at the time of this study; they are now ex‐employees.

## Supporting information


**Data S1:** ejh70032‐sup‐0001‐supinfo.docx.

## Data Availability

The data are not publicly available due to privacy or ethical restrictions. All data relevant to the study are included in the article or uploaded as Supporting Information.
