# Supplementary material for: Outcomes and Healthcare Resource Utilisation in Adults With von Willebrand Disease Receiving On‐Demand Recombinant von Willebrand Factor in the United Kingdom
Source: Eur J Haematol. 2025 Oct 10;116(1):66–74. doi: 10.1111/ejh.70032 (PMC12673354; doi:10.1111/ejh.70032)
Supplement: Supplementary file 1 — Data S1: ejh70032‐sup‐0001‐supinfo.docx. [file EJH-116-66-s001.docx]

**Outcomes and Healthcare Resource Utilisation in Adults with von Willebrand Disease Receiving On-demand Recombinant von Willebrand Factor in the United Kingdom**

*Authors:* Mike Laffan^1^, Heena Howitt^2^, Cheryl Jones^3^, Sarah Brighton^3^, Rosa Willock^3^, Anna Sanigorska^3^, Oliver Heard^2^

*Author affiliations:*

^1^Centre for Haematology, Imperial College London, Hammersmith Hospital, London, UK

^2^Takeda UK Ltd., London, UK

^3^HCD Economics, Knutsford, UK

*Correspondence details:*

Name: Mike Laffan

Address: Centre for Haematology, Imperial College London

Hammersmith Hospital

London

W12 ONN

Telephone: +44 7788 106057

Email: [m.laffan@imperial.ac.uk](mailto:m.laffan@imperial.ac.uk)

**Supporting Information**

**Table S1** Demographics and baseline characteristics of all adults who experienced a spontaneous or traumatic or surgery-related bleed that was treated with rVWF at index

| **Characteristic** | **N=32** |
| --- | --- |
| Age at index (years), mean (SD)  Age groups at index, n (%)  18–29 years  30–44 years  45–64 years  ≥65 years | 48.3 (19.5)  5 (15.6)  13 (40.6)  5 (15.6)  9 (28.1) |
| Female, n (%)  White/Caucasian, n (%) | 22 (68.8)  29 (90.6) |
| Weight (kg), mean (SD)  BMI (kg/m^2^), mean (SD)  VWD type, n (%)  Type 1  Type 2A  Type 2B  Type 2M  Type 2N  Unclassified  Age at diagnosis (years), mean (SD)  Time since diagnosis (years), mean (SD)  Familial history of VWD, n (%)  Unknown, n (%)  Family member with VWD, n (%)^†^  Parent  Sibling  Grandparent  Other  No history of GI bleeding, n (%)  Laboratory test values at diagnosis (IU/ml)^‡^, mean (SD)  VWF:RCo [n=18]  FVIII:C [n=18]  VWF:Ag [n=19] | 82.6 (18.6)  28.7 (5.5)  9 (28.1)  6 (18.8)  7 (21.9)  7 (21.9)  1 (3.1)  2 (6.3)  27.0 (20.3)  20.8 (15.8)  17 (53.1)  11 (34.4)  13 (76.5)  8 (47.1)  4 (23.5)  9 (52.9)  30 (93.8)  0.2 (0.2)  0.6 (0.3)  0.2 (0.1) |
| Comorbidities at index^§^, n (%)  0  1  2  >2  Comorbidities at index by type^¶^, n (%)  Disease of the musculoskeletal system or connective tissue  Circulatory  Disease of digestive system  Respiratory & ENT  Autoimmune  Mental or behavioural disorder  Oncology  Other  Cardiovascular  Endocrine, nutritional or metabolic disease  Neurological  Obstetrics/gynaecology  Disease of the blood/blood-forming organs  Disease of the genitourinary system  Liver disease  Ophthalmology | 13 (40.6)  6 (18.8)  4 (12.5)  9 (28.1)  8 (25.0)  6 (18.8)  6 (18.8)  4 (12.5)  3 (9.4)  3 (9.4)  3 (9.4)  3 (9.4)  2 (6.3)  2 (6.3)  2 (6.3)  2 (6.3)  1 (3.1)  1 (3.1)  1 (3.1)  1 (3.1) |

N=number of patients.

^†^Percentage calculated with number of patients with non-missing data in denominator.

^‡^Not all lab tests were performed at diagnosis on all patients. Mean (SD) are reported only for patients on whom each test was performed [n=x].

^§^Within two years of data abstraction.

^¶^At least one comorbidity in category.

Ag, antigen; BMI, body mass index; ENT, ear, nose and throat; FVIII, factor VIII; GI, gastrointestinal; RCo, ristocetin cofactor; rVWF, recombinant von Willebrand factor; SD, standard deviation; VWD, von Willebrand disease; VWF, von Willebrand factor

**Table S2** Laboratory test values at presentation for adults who experienced a spontaneous or traumatic bleed that was treated on demand with rVWF at index by VWD type and by bleed severity

|  | **VWF:RCo  IU/mL** | **FVIII:C IU/mL** | **VWF:Ag IU/mL** |
| --- | --- | --- | --- |
| VWD type |  |  |  |
| Type 1 | [n=0] | 0.6 (N/A) [n=1] | 0.3 (N/A) [n=1] |
| Type 2 | 0.4 (0.3) [n=7] | 1.1 (0.8) [n=7] | 0.8 (0.8) [n=7] |
| Bleed severity |  |  |  |
| Mild | 0.5 (0.4) [n=4] | 1.4 (0.8) [n=5] | 1.0 (0.9) [n=5] |
| Moderate | 0.1 (0.0) [n=2] | 0.5 (0.3) [n=2] | 0.3 (0.2) [n=2] |
| Severe | 0.1 (N/A) [n=1] | 0.6 (N/A) [n=1] | 0.6 (N/A) [n=1] |

All data are presented as mean (SD).

Not all lab tests were performed at presentation on all patients. Mean (SD) are reported only for patients on whom each test was performed [n=x].

Ag, antigen; FVIII, factor VIII; N/A, not applicable; RCo, ristocetin cofactor; rVWF, recombinant von Willebrand factor; SD, standard deviation; VWD, von Willebrand disease; VWF, von Willebrand factor.

**Table S3** On-demand treatment of bleed events at index, pre- and post-index in adults with VWD by treatment

| **Treatment regimens/combinations, n (%)** | **Pre-index**  **(N=5)** | **Index**  **(N=12)** | **Post-index**  **(N=13)** | **Total**  **(N=30)** |
| --- | --- | --- | --- | --- |
| **rVWF only**  **rVWF plus other treatment(s)**  rVWF + TXA  rVWF + pdVWF/FVIII complex + TXA  rVWF + FVIII + TXA | **0 (0.0)**  **0 (0.0)**  0 (0.0)  0 (0.0)  0 (0.0) | **4 (33.3)**  **6 (50.0)**  5 (41.7)  1 (8.3)  0 (0.0) | **2 (15.4)**  **8 (61.6)**  5 (38.5)  2 (15.4)  1 (7.7) | **6 (20.0)**  **14 (46.7)**  10 (33.3)  3 (10.0)  1 (3.3) |
| **Other VWF treatments**  pdVWF/FVIII complex  pdVWF/FVIII complex + TXA | **3 (60.0)**  1 (20.0)  2 (40.0) | **0 (0.0)**  0 (0.0)  0 (0.0) | **1 (7.7)**  0 (0.0)  1 (7.7) | **4 (13.3)**  1 (3.3)  3 (10.0) |
| **Treatment switches**  From rFVIII + TXA to rVWF + TXA  From pdVWF/FVIII complex to rVWF + TXA  From pdVWF/FVIII complex to rVWF | **0 (0.0)**  0 (0.0)  0 (0.0)  0 (0.0) | **2 (16.7)**  1 (8.3)  1 (8.3)  0 (0.0) | **1 (7.7)**  0 (0.0)  0 (0.0)  1 (7.7) | **3 (10.0)**  1 (3.3)  1 (3.3)  1 (3.3) |
| **No treatment** | **2 (40.0)** | **0 (0.0)** | **1 (7.7)** | **3 (10.0)** |

FVIII, factor VIII; pd, plasma-derived; rFVIII, recombinant FVIII; rVWF, recombinant von Willebrand factor; SD, standard deviation; TXA, tranexamic acid; VWD, von Willebrand disease; VWF, von Willebrand factor

**Table S4** On-demand treatment of spontaneous and traumatic bleeds pre-index in adults (N=5) with VWD by treatment

| **Medication** | **pdVWF/FVIII complex (IU)** | **Tranexamic acid  (mg)** |
| --- | --- | --- |
| **Variable**  Number of bleed events, n (%) | 3 (100.0) | 2 (100.0) |
| Dose (IU or mg), mean (SD)  Number of infusions  Dose per infusion (IU/kg or mg/kg)  Total consumption (IU or mg)  Duration of treatment (days) | 1000.0 (0.0)  1.0 (0.0)  10.9 (0.7)  1000.0 (0.0)  1.0 (0.0) | 1000 (0.0)  12.0 (12.7)  11.4 (0.0)  12,000.0 (12,727.9)  4.5 (3.5) |

FVIII, factor VIII; pd, plasma-derived; SD, standard deviation; VWD, von Willebrand disease; VWF, von Willebrand factor.

**Table S5** Post-index spontaneous and traumatic bleed outcomes in adults (N=13) with VWD by on-demand treatment

| **Medication** | **rVWF (IU)**  **(N=11)** | **pdVWF/FVIII complex (IU) (N=4)** | **FVIII (IU)**  **(N=1)** | **Tranexamic acid (mg)**  **(N=9)** |
| --- | --- | --- | --- | --- |
| **Outcome, n (%)**  Bleed resolution  Yes  No  Unknown | 8 (72.7)  2 (18.2)  1 (9.1) | 2 (50.0)  2 (50.0)  0 (0.0) | 1 (100.0)  0 (0.0)  0 (0.0) | 8 (88.9)  1 (11.1)  0 (0.0) |
| Bleed control  Yes  No  Partly | 9 (81.8)  0 (0.0)  2 (18.2) | 1 (25.0)  2 (50.0)  1 (25.0) | 0 (0.0)  0 (0.0  1 (100.0) | 1 (11.1)  1 (11.1)  7 (77.8) |
| Treatment switch  Yes  No | 0 (0.0)  11 (100.0) | 1 25.0)  3 (75.0) | 0 (0.0)  1 (100.0) | 0 (0.0)  9 (100.0) |

FVIII, factor VIII; pd, plasma-derived; rVWF, recombinant von Willebrand factor; SD, standard deviation; VWD, von Willebrand disease; VWF, von Willebrand factor.

**Section S1: Differences in on-demand treatment and bleed-related outcomes for pooled index, pre-index, and post-index bleed events treated with rVWF only, or with rVWF plus another treatment in adults with VWD**

It was assumed that treatments were used concomitantly if they were used to treat the same bleed, unless indicated by a treatment switch. **Table S1** summarises treatments used, alone or in combination, to treat bleed events during the pre-index, index, and post-index periods. Mean (SD) total consumption of rVWF was lower for bleeds treated with rVWF only compared with those treated with rVWF plus another treatment (3575.0 [1827.0] vs. 4407.1 [4263.9] IU per patient, respectively). However, the mean (SD) dose per patient per kg was higher for patients treated with rVWF only compared with those treated with rVWF plus another treatment (35.6 [15.3] vs. 32.3 [12.3] IU/kg, respectively).

In terms of treatment outcomes, patients treated with rVWF only reported 100% bleed resolution and bleed control. Patients treated with rVWF plus another treatment reported bleed resolution for 78.6% of bleeds and bleed control for 85.7% of bleeds. For all bleed events treated with on-demand rVWF only, the treating physicians rated treatment satisfaction excellent (33.3%) or good (66.7%). For all bleed events treated with on-demand rVWF plus another treatment, all physicians rated treatment satisfaction excellent (100.0%).

**Section S2: Stratified analyses**

***Age group***

Spontaneous and traumatic bleeds were most commonly recorded in the 30–44 years age group (**Table S5**), with the most common bleed types being pregnancy-related (30.8%) and menorrhagia (23.1%). These were also the most commonly recorded bleed types in the 18–29 years age group (33.3% each).

**Table S6** Spontaneous and traumatic bleeds treated on demand with rVWF pooled across index, pre-index, and post-index by age group

| **Age group, n (%)** | **Bleeds (N=23)** |
| --- | --- |
| 18–29 years | 6 (26.1) |
| 30–44 years | 13 (56.5) |
| 45–64 years | 2 (8.7) |
| 65+ years | 2 (8.7) |

The mean (SD) dose of rVWF per infusion used to treat bleeds was highest in the 18–29 years age group (41.3 [7.7] IU/kg), followed by the 30–44 years age group (31.3 [12.6] IU/kg. The older age groups, 45–64 years and 65+ years, received mean (SD) 24.5 [22.1] and 24.6 [6.5] IU/kg, respectively. Bleed resolution with rVWF treatment was recorded for 5 of 6 bleed events in the 18–29 years age group (1 remaining bleed event was recorded as bleed resolution unknown, bleed control achieved), and for 11 of 13 bleed events in the 30–44 years age group (both remaining bleed events were recorded as no bleed resolution, partial bleed control achieved). rVWF treatment satisfaction was rated as good or excellent by the treating physician for all bleeds, with excellent most commonly reported for bleeds in those aged 30–44 (92.3%). Inpatient stays were most common in the 65+ years age group (100.0%) followed by the 30–44 years age group (46.2%), and day cases were most common in the 45–64 years age group (100.0%).

***Sex***

Spontaneous and traumatic bleeds were more commonly reported in females (21 [91.3%]), compared with males (2 [8.7%]), with pregnancy-related bleeds (28.6%) and menorrhagia (23.8%) being the most frequently observed bleed types in females. All rVWF-treated bleeds recorded in males were a result of trauma (100.0%). The mean (SD) dose of rVWF per infusion used to treat bleeds was higher in females (33.7 [12.7] IU/kg) than in males (23.1 [4.3] IU/kg). However, the mean (SD) number of infusions and treatment duration were both higher in male patients (7.5 [6.4] infusions, 4.5 [2.1] days) than in female patients (1.7 [2.0] infusions, 1.5 [1.0] days). Bleed resolution with rVWF treatment was recorded for 18 of 21 bleed events in females (1 bleed event was recorded as bleed resolution unknown and 2 bleed events were recorded as no bleed resolution). rVWF treatment satisfaction was rated as good or excellent by the treating physician for all bleeds, with excellent reported for 100.0% of bleeds in males and 81.0% in females. Inpatient stays were recorded for 100.0% of rVWF-treated bleeds in males and 38.1% in females; day cases were recorded for 61.9% of bleeds in females.

***VWD type***

Spontaneous and traumatic bleeds were most commonly reported in patients with type 2 VWD (91.3%), with 8.7% of bleed events in patients with type 1 VWD. The bleed type most frequently observed in type 2 VWD patients was a pregnancy-related bleed (28.6%), followed by menorrhagia (23.8%). rVWF-treated bleeds recorded in type 1 patients were a muscle haematoma and a gastrointestinal bleed. The mean (SD) dose of rVWF per infusion used to treat bleeds was higher in type 1 VWD patients (44.4 [6.1] IU/kg) than in type 2 VWD patients (31.6 [12.5] IU/kg). Bleed resolution with rVWF treatment was recorded for 18 of 21 bleeds in type 2 VWD patients (1 bleed event was recorded as bleed resolution unknown and 2 bleed events were recorded as no bleed resolution). rVWF treatment satisfaction was rated as good or excellent by the treating physician for all bleeds (100.0%), with excellent reported for 90.5% of bleeds in type 2 VWD patients. Inpatient stays were recorded for 47.6% of rVWF-treated bleeds in type 2 VWD patients. Day cases were recorded for 100.0% of bleeds in type 1 VWD patients and 52.4% of bleeds in type 2 VWD patients.

***Time since diagnosis***

Of the rVWF-treated spontaneous and traumatic bleed events captured in the study period, 15 (65.2%) were reported in patients with a time since diagnosis of more than 10 years, whereas 8 (34.8%) were reported in patients within 0–10 years after diagnosis. The bleed types most frequently observed in those diagnosed with VWD more than 10 years prior were pregnancy-related bleeds (26.7%) and menorrhagia (26.7%). The most commonly reported types of rVWF-treated bleed recorded in those diagnosed 0–10 years prior were pregnancy-related bleeds (25.0%) and muscle haematomas (25.0%). The mean (SD) dose of rVWF per infusion used to treat bleeds was higher in patients diagnosed more than 10 years prior (35.9 [10.7] IU/kg) than those diagnosed 0–10 years prior (26.9 [14.3] IU/kg). Bleed resolution with rVWF treatment was recorded for all bleeds (100%) in patients diagnosed 0–10 years prior, and for 12 of 15 bleeds in patients diagnosed more than 10 years prior (1 bleed event was recorded as bleed resolution unknown and 2 bleed events were recorded as no bleed resolution). rVWF treatment satisfaction was rated as good or excellent by the treating physician for all bleeds, with excellent reported for 75.0% of bleeds in patients diagnosed 0–10 years prior, and 86.7% in those diagnosed more than 10 years prior. Inpatient stays were recorded for 37.5% of rVWF-treated bleeds in patients within the first 10 years post-diagnosis, and for 46.7% of bleeds in those diagnosed more than 10 years prior. Day cases were recorded for 50.0% and 60.0% of rVWF-treated bleeds, respectively.

***Number of comorbidities***

Spontaneous and traumatic bleeds were most commonly recorded in patients with no comorbidities (**Table S6**), with the most common bleed types being pregnancy-related (50.0%) and menorrhagia (20.0%). Menorrhagia was also common in patients with one comorbidity (42.9%).

**Table S7** Spontaneous and traumatic bleeds treated on demand with rVWF pooled across index, pre-index, and post-index by number of comorbidities

| **Number of comorbidities, n (%)** | **Bleeds (N=23)** |
| --- | --- |
| 0 | 10 (43.5) |
| 1 | 7 (30.4) |
| 2 | 4 (17.4) |
| 3+ | 1. (8.7) |

The mean (SD) dose of rVWF per infusion used to treat bleeds was highest in patients with one comorbidity (39.3 [11.6] IU/kg), followed by those with no comorbidities (31.8 [15.0] IU/kg). Patient groups with multiple comorbidities received mean (SD) 27.7 [3.7] IU/kg (two comorbidities) and 24.6 [6.5] IU/kg (three or more comorbidities). Bleed resolution with rVWF treatment was recorded for 9 of 10 bleeds in the zero comorbidities group (1 bleed event was recorded as bleed resolution unknown), and 5 of 7 bleeds in the group with one comorbidity (two bleed events were recorded as no bleed resolution). rVWF treatment satisfaction was rated as good or excellent by the treating physician for all bleeds, with excellent most commonly reported for bleeds in those with two comorbidities (100.0%), and for bleeds in those with no comorbidities (90.0%). Inpatient stays were most commonly required in the group with three or more comorbidities (100.0%), followed by those with no comorbidities (50.0%). Day cases were most common for bleeds occurring in patients with two comorbidities (100.0%), and for bleeds occurring in patients with one comorbidity (71.4%).

***Bleed severity***

Of the rVWF-treated spontaneous and traumatic bleed events captured in the study period, 14 (60.9%) were mild, 5 (21.7%) were moderate, and 4 (17.4%) were severe. The bleed type most frequently observed for mild bleeds was a pregnancy-related bleed (35.7%), followed by a traumatic bleed (21.4%). Epistaxis was the most common type of moderate bleed (40.0%), and menorrhagia was the most common type of severe bleed (75.0%). The mean (SD) dose of rVWF per infusion used to treat bleeds was highest for severe bleeds (39.2 [5.2] IU/kg), and lowest for mild bleeds (30.3 [13.7] IU/kg). Moderate bleeds were treated with mean (SD) 34.5 (13.0) IU/kg per infusion. Bleed resolution with rVWF treatment was recorded for 13 of 14 mild bleeds (one bleed event was recorded as bleed resolution unknown), all moderate bleeds, and 2 of 4 severe bleeds (two bleed events were recorded as no bleed resolution). rVWF treatment satisfaction was rated as good or excellent by the treating physician for all bleeds, with excellent most commonly reported for moderate bleeds (100.0%) compared to 78.6% of mild and 75.0% of severe bleeds. Inpatient stays were most frequently required for severe bleeds (75.0%), and day cases were most common for moderate bleeds (80.0%).
